# Identification of Novel Yellow Fever Class II Epitopes in YF-17D Vaccinees

**DOI:** 10.3390/v12111300

**Published:** 2020-11-12

**Authors:** Jose Mateus, Alba Grifoni, Hannah Voic, Michael A. Angelo, Elizabeth Phillips, Simon Mallal, John Sidney, Alessandro Sette, Daniela Weiskopf

**Affiliations:** 1 Center for Infectious Disease and Vaccine Research, La Jolla Institute for Immunology (LJI), La Jolla, CA 92037, USA; jmtrivino@lji.org (J.M.); agrifoni@lji.org (A.G.); hvoic@lji.org (H.V.); mikey.angelo88@gmail.com (M.A.A.); jsidney@lji.org (J.S.); 2 Institute for Immunology and Infectious Diseases, Murdoch University, Perth, WA 6150, Australia; E.Phillips@murdoch.edu.au (E.P.); S.Mallal@murdoch.edu.au (S.M.); 3 Department of Medicine, Division of Infectious Diseases and Global Public Health, University of California, San Diego (UCSD), La Jolla, CA 92037, USA

**Keywords:** yellow fever virus, epitopes, CD4^+^ T cells, vaccination, T cells

## Abstract

Yellow fever virus (YFV) is a mosquito-borne member of the genus flavivirus, including other important human-pathogenic viruses, such as dengue, Japanese encephalitis, and Zika. Herein, we report identifying 129 YFV Class II epitopes in donors vaccinated with the live attenuated YFV vaccine (YFV-17D). A total of 1156 peptides predicted to bind 17 different common HLA-DRB1 allelic variants were tested using IFNγ ELISPOT assays in vitro re-stimulated peripheral blood mononuclear cells from twenty-six vaccinees. Overall, we detected responses against 215 YFV epitopes. We found that the capsid and envelope proteins, as well as the non-structural (NS) proteins NS3 and NS5, were the most targeted proteins by CD4^+^ T cells from YF-VAX vaccinated donors. In addition, we designed and validated by flow cytometry a CD4^+^ mega pool (MP) composed of structural and non-structural epitopes in an independent cohort of vaccinated donors. Overall, this study provides a comprehensive prediction and validation of YFV epitopes in a cohort of YF-17D vaccinated individuals. With the design of a CD4 epitope MP, we further provide a useful tool to detect ex vivo responses of YFV-specific CD4 T cells in small sample volumes.

## 1. Introduction

Yellow fever (YF) is a mosquito-borne acute viral disease, currently endemic to the tropical and subtropical regions of Africa and the Americas. Although many people do not experience symptoms, the most severe cases include acute fever, organ failure, jaundice, dark urine, abdominal pain, and even death [1]. YF is caused by the yellow fever virus (YFV), an RNA virus from the flavivirus genus, which is closely related to the dengue, Japanese encephalitis, Zika, and tick-borne encephalitis viruses [2]. The *Aedes* or *Haemagogus* species of mosquitoes, are responsible for YFV transmission through sylvatic, intermediate, and urban cycles, affecting both human and non-human primates. Before introducing large-scale vaccination, the impact of YF epidemics on human health, especially amongst children and the elderly, was staggering [3]. Development of the live attenuated YF-17D based vaccines (17D-204 and 17DD) in the late 1930s and subsequent large-scale immunization programs, led to the control and virtual elimination of urban epidemics. The yellow fever 17D-based vaccine (YF-17D) is a live attenuated vaccine produced by Sanofi Pasteur under the tradename YF-VAX® and is the only YF vaccine licensed in the United States [4].

Vaccination is an important strategy to combat flaviviral diseases, and the YF vaccine is amongst the most successful vaccines ever produced [5]. A single dose of the YF vaccine elicits neutralizing antibodies in vaccinated individuals for 30 or more years, and may provide lifelong immunity [6]. Several studies have reported class II and class I restricted epitopes [7,8,9,10,11,12,13,14,15,16,17,18,19], but a systematic analysis providing comprehensive coverage of common HLA types has not been reported. The interest of such an analysis is several-fold. 

First, T cell responses are an essential component of the immune responses elicited YFV. However, in contrast to multiple system biology studies that analyzed correlates of protection at the level of antibody responses [6,20,21], T cell responses as potential correlates of the protection and immunity elicited YFV vaccination are not fully understood [22], despite circumstantial evidence that suggests that T cell responses are important for immunity to flaviviruses in general and to YFV in particular [22,23,24]. 

Second, a detailed assessment of T cell responses to YF in the natural setting, is also important in the context of potential disease outbreaks, to define natural disease, potential immunopathology, and host-virus interactions. These are pressing concerns because, despite the success and efficacy of the YF-17D vaccine, the World Health Organization in 2018 reported major YF outbreaks in five countries (Brazil, Congo, Ethiopia, Nigeria, and South Sudan), cases with epidemic potential were informed from the Democratic Republic of the Congo, and signals of disease activity were reported in Central African Republic, Guinea, Liberia, Bolivia, Colombia, French Guiana, and Peru [1]. Due to multiple factors, such as low vaccination coverage against YFV in individuals living in endemic areas, the presence of non-human wildlife reservoirs, and the climate change-related expansion of suitable endemic areas in previously non-endemic regions, there is a high probability of future YF epidemics [25,26,27]. 

Third, it is important to be able to characterize YF-induced T cell responses, to be able to assess whether these responses are cross-reactive with other flaviviruses, and because YF backbones are used as a vaccine delivery vector [24]. Considering different flaviviruses species, little cross-reactivity exists at the level of antibody responses that target mostly the E protein, while T cell responses are known to target Non-structural (NS) proteins, which are more conserved, raising the possibility that T cell responses might be more cross-reactive. Whether cross-reactive T cell responses can alter or influence the outcome of infection with other related flaviviruses is currently undetermined. It is also important to assess cross-reactivity in light of the fact that YFV is also utilized as a carrier for delivery of antigens from other pathogens, as illustrated by the DENGVAXIA vaccine, which utilizes a YFV backbone to deliver pre-membrane (prM) and envelope (E) proteins from the four serotypes of dengue virus [28]. The use of YF vaccination to induce or recall responses against specific epitopes is, therefore, of interest from various perspectives.

In the present study, we analyzed the human CD4^+^ T cell responses and associated epitope repertoires restricted by HLA class II in vaccinated individuals. Peptides restricted by 17 HLA Class II DRB1 alleles were synthesized and tested for their capacity to recall T cell responses from peripheral blood mononuclear cells (PBMCs) derived from HLA-matched donors that received the YF vaccine. Based on the results of this epitope identification screen, we designed and validated a pool of YF-derived CD4 epitopes that allows the ex vivo detection of vaccine-induced CD4^+^ T cell responses in small sample volumes, irrespective of HLA phenotype. 

## 2. Materials and Methods

### 2.1. Human Blood Samples from Vaccinated Individuals

For the epitope identification studies, twenty-six peripheral blood samples were obtained from local adult blood donors from the San Diego area in an anonymous fashion. Donors previously vaccinated with YF-VAX included in the present study, were of both genders, and aged between 18 and 59 years old. Samples were collected over 40 months, between April 2015 and August 2018. The characteristics of this study population are summarized in Table S1. 

To confirm the identified epitopes and the mega pool validation, we utilized 15 peripheral blood samples from previously YFV vaccinated volunteers obtained from local adult blood donors from the San Diego area in an anonymous fashion. Moreover, in this cohort, both sexes were represented, and donors ranged from 18 to 60 years of age. Details of this donor cohort are shown in Table S2.

All donors utilized in this study have been collected in the greater San Diego area where neither dengue, Japan encephalitis, or Zika viruses are endemic. However, we cannot exclude that any of the study participants have traveled to endemic areas and could, thus, have been exposed to other flaviviruses.

Peripheral blood mononuclear cells (PBMCs) were purified by density gradient centrifugation (Ficoll-Paque Premium; GE Healthcare Biosciences) resuspended in fetal bovine serum (Gemini Bio-products, Sacramento, CA) containing 10% dimethyl sulfoxide (DMSO), and subsequently cryopreserved in liquid nitrogen. Institutional Review Board (IRB) of La Jolla Institute for Immunology approved all protocols described in the present study (VD-101).

### 2.2. HLA Typing

Donors for the epitope identification studies were HLA typed by an ASHI-accredited laboratory at Murdoch University (Western Australia) using locus-specific PCR amplification on genomic DNA as previously described [29]. Donors and their corresponding HLA phenotype are listed in Table S1.

### 2.3. MHC Class II Binding Predictions and Peptide Selection 

A panel of 681 15-mer peptides overlapping by 10 residues and spanning the entirety of the YF-17D polyprotein was synthesized (Mimotopes, Victoria, Australia) on a small scale as crude material. Each peptide was then submitted for analysis to identify the top 10% (*n* = 68) scoring peptides for each HLA class II considered. HLA-DRB1 binding predictions were performed using the Immune Epitope Database (IEDB), and Analysis Resource (www.iedb.org; [30]) recommended methods, as of July 2016 [31,32,33]. A panel of 17 HLA-DRB1 alleles was considered, to include, DRB1*01:01, DRB1*03:01, DRB1*04:01, DRB1*04:03, DRB1*04:05, DRB1*04:07, DRB1*07:01, DRB1*08:02, DRB1*10:01, DRB1*11:01, DRB1*11:04, DRB1*12:01, DRB1*13:01, DRB1*15:01, DRB1*15:02, DRB1*15:06, and DRB1*16:01. For each allele, the top 68 scoring peptides were further divided into seven pools of approximately 10 peptides each, to be used in the epitope screening experiments. 

### 2.4. In Vitro Expansion of YFV-Specific T Cells

CD4+ T cells were isolated by magnetic bead negative selection (Miltenyi Biotech, Bergisch Gladbach, Germany) and co-cultured with autologous antigen presenting cells (APCs) at a 2:1 ratio in RPMI 1640 (Omega Scientific, Tarzana, CA) supplemented with 5% human serum (Cellgro, Lincoln, NE,) at a concentration of 4 × 106 cells/mL in 24-well plates (BD Biosciences, San Diego, CA). Cells were stimulated with YF-specific DRB1 pools [1 μg/mL] and additional IL-2 [10 U/mL]; eBioscience was added every four days after the initial pool-stimulation, as previously described [34]. After 14 days of in vitro expansion, the IFNγ response against individual peptides was evaluated by ELISPOT assay.

### 2.5. Ex Vivo IFNγ ELISPOT Assay

PBMCs (2 × 10^5^ cells/well) were incubated in triplicates with 2 μg/mL of HLA-matched peptides in plates coated with the anti-human IFNγ antibody (mAb 7-B6-1; Mabtech, Stockholm, Sweden) at 37 °C for 20–24 h and developed as previously described [34,35]. If the rare event when the exact HLA match was not available, we considered closely related HLA alleles within the same supertype [36]. PBMCs were first screened with peptide pools containing 10 peptides per pool and subsequently deconvoluted to identify the individual epitopes [37].

### 2.6. YFV-CD4 Megapool Design and Manufacture

To design a Megapool (MP) encompassing epitopes providing maximum coverage and breadth, the set of epitopes experimentally defined in this study was expanded to include additional epitopes independently identified in other studies, and predicted promiscuous epitopes as previously reported [35,38]. First, we queried the IEDB (April, 2017) for previously identified YFV CD4 epitopes using the following query parameters: Assay-Positive Assays Only; Assay-T Cell Assays (i.e., no B Cell Assays or MHC Ligand Assays); Antigen/Organism: Yellow fever virus (ID:11089); Host-Humans; and MHC restriction: MHC Class II. The resulting set composed of 94 epitopes was further supplemented by additional prediction analyses. Prediction of HLA class II promiscuous binding peptides was carried out using sequences from the YF-17D vaccine strain and virus isolates derived from a recent Brazilian outbreak (protein ID: ARM37843.1) [39] by using the “7-allele-method” [40] embedded in the Tepitool tool [41], considering peptides with a median consensus percentile cutoff of ≤20. To avoid redundancy across and within the three different datasets, epitopes were clustered using the IEDB cluster 2.0 tool [42] applying the clique clustering method and 70% homology criteria. Overall, the experimentally defined YFV epitopes of this study (*n* = 129) were combined with the IEDB derived epitopes (*n* = 94) and the ones retrieved from the prediction (*n* = 292) for a CD4 YFV-MP containing, after clustering, a total of 275 peptides (Table S3). These 275 peptides were synthesized, and an epitope MP was derived as previously described by sequential lyophilization. The resulting MP lyocake was resuspended in DMSO to a concentration of 2 mg/mL of each individual peptide as previously described [38].

### 2.7. Flow Cytometry

The following conjugated Abs were used in the present study: CD3-Alexa Fluor 700 (OKT3), CD8-BV650 (RPA-T8) (Biolegend, San Diego, CA, USA), CD4-APC-efluor 780 (RPA-T4), IFNγ-FITC (4S.B3) (Thermo Fisher Scientific, Waltham, MA, USA), CD14-V500 (M5E2), and CD19-V500 (HIB19) (BD Biosciences; San Jose, CA, USA). For the exclusion of dead cells, the Fixable Viability Dye eFluor™ 506 (LIVE/DEAD) was used (Thermo Fisher Scientific). To identify the IFNγ production on antigen-specific CD4^+^ T cells, one million of PBMCs were cultured with CD4^+^ YFV-MP (1 μg/mL), Dimethyl sulfoxide (DMSO) (1%) as a negative control, and Phorbol 12-Myristate 13-Acetate (PMA) (0.1 μg/mL) and ionomycin (1 μg/mL) as a positive control in the presence of brefeldin A (1 μg/mL) (BD Biosciences) for 6 h and subsequently permeabilized and stained as described previously [35]. Cells from donors have been excluded from the analysis if the IFNγ response to PMA and ionomycin stimulation was lower than 1% in the CD3^+^ cells. Cells were acquired on an LSR-II flow cytometer (BD Biosciences), and the analysis was performed using FlowJo 10.5.3. software (BD Biosciences).

### 2.8. Statistical Analysis

All statistical analyses were performed using the Wilcoxon-rank test. The test was two-tailed, and statistical significance was achieved at *p* < 0.05. GraphPad Prism 8.0 software (San Diego, CA, USA) was used for statistical analyses.

## 3. Results

### 3.1. Selection of a Panel of DRB1 Alleles that Affords High Population Coverage

Our goal was to identify a comprehensive set of Yellow Fever epitopes by assessing the CD4^+^ T cell response in a cohort of vaccinated donors with YF-VAX®. Table S1 lists the gender, age, months post-vaccination, and HLA typing for each donor. To ensure that our donor cohort was representative of the general population, we compared frequencies of the main HLA class II alleles observed in our donor cohort with those observed in our repository of over 3500 donors, reflecting a variety of clinical studies and a diverse set of ethnicities, ranging from the USA, South and Central America, Asia, South Africa, and Europe (Figure S1A). 

In general, the HLA representation was similar in the different populations. Of the eleven different HLA class II alleles identified in our cohort with phenotypic frequencies >5%, seven (64%) are also present in the general population with frequencies >5%. At the same time, nine (82%) of the eleven DRB1 alleles included in a reference panel of the most common and representative class II alleles in the general population [43] are also present in the cohort studied here. The three most common DRB1 alleles in the cohort (DRB1*04:01, DRB1*13:01, and DRB1*15:01), present with frequencies of 15% or greater, are also found in the worldwide population with frequencies >5%. 

Next, we selected for our experiments a panel of seventeen different DRB1 alleles (see methods for list) based on their being represented in the donor cohort, and also being reasonably frequent worldwide. Figure S1B shows that the seventeen HLA-DRB1 alleles selected allow coverage of both alleles in 65% of the individuals from the donor cohort from Table S1, and for one allele in the remaining 35%; all donors are represented by at least one allele in the prediction panel. The same analysis with the general repository of over 3500 donors, reflecting a variety of clinical studies and a diverse set of ethnicities, ranging from the USA, South and Central America, Asia, South Africa, and Europe, found similar representation by the panel, with 51% of individuals covered for both alleles, and 38% by one; only 11% were not covered at all. 

### 3.2. Identification of Yellow Fever Epitopes

A total of 681 fifteen-mer peptides overlapping by 10 residues spanning the entirety of the YF-17D polyproteins were synthesized. Next, the Analysis Resource from IEDB was used to predict the capacity of these 681 peptides to bind the selected HLA-DRB1 alleles. Sets of the top 10% binders (corresponding to 68 peptides) were then selected for each allele, and tested in donors expressing the matching HLA-DRB1 variant.

Each of the peptide sets was screened in 1–8 (average 2.8) HLA-matched donors. If the rare event when the exact HLA match was not available, we considered closely related HLA alleles within the same supertype [36]. A total of 215 epitopes, representing 129 unique epitope sequences, were identified; 69 of these are described here for the first time. The top 40 epitopes, which account for 75% of the total response, are listed in Table 1, which also shows the number of responding donors, and putative HLA restriction; overall, 90% of the total response can be attributed to the top 70 epitopes. Table S4 shows a complete listing of all peptides identified, including response frequency and HLA restriction.

### 3.3. CD4^+^ T Cells Recognize both Structural and Non-Structural Proteins 

**Figure 1:**
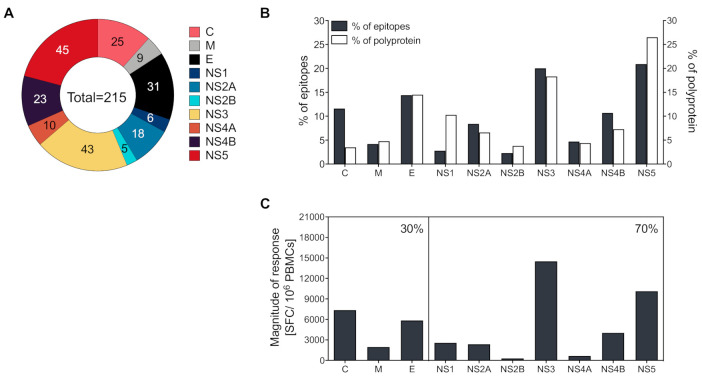
Protein location and magnitude of response of CD4^+^ epitopes identified. (**A**) Patterns of protein immunodominance of CD4^+^ T cells responses against yellow fever virus (YFV) epitopes. The fraction of identified epitopes is plotted as a function of the protein they are derived from (capsid protein [C], membrane protein [M], envelope protein [E], non-structural protein 1 [NS1], NS2A, NS2B, NS3, NS4A, NS4B, and NS5). Numbers represent the number of epitopes identified. (**B**) Relative distribution of epitopes derived from the structural (C, M, and E) or NS proteins (NS1–5). The gray bars reflect a relative number of peptides for each protein predicted to bind the various HLA-DR molecules, and the white bars show the percentage of all possible peptides (percentage of the YF-17D polyproteins) accounted for by each antigen. (**C**) The magnitude of response per structural (C, M, and E) or NS proteins (NS1–5). The magnitude of response was defined as the average sum total of spot-forming cells (SFCs)/donor associated with each protein. Percentages in the upper right corner reflect the relative response directed at either structural or NS protein responses.

Epitopes were derived from all three structural proteins, as well as the seven non-structural proteins (NS1, NS2A, NS2B, NS3, NS4A, NS4B, and NS5). The structural proteins C and E, as well as the non-structural proteins NS3 and NS5, were the most dominantly targeted CD4 antigens and accounted for 25, 31, 43, and 45 of the 215 total epitopes, respectively (Figure 1A). 

Next, we evaluated if antigen size affects the CD4 targeting preference. While there is a correlation between the size and percent of CD4 epitopes identified for M, E, NS2A, NS3, NS4, a clear exception is represented by the dominance of the C antigen, which is more immunogenic than size alone would predict (Figure 1B). When the magnitude of response was evaluated, C, E, NS3, and NS5 were the most immunogenic antigens, accounting for 15, 12, 29, and 20% of the total magnitude of response, respectively. In total, 30% of the response was associated with structural proteins and 70% with non-structural proteins (Figure 1C).

### 3.4. Magnitude of Responses Varies as a Function of HLA

The overall immunoreactivity as a function of the different HLA-DRB1 allelic peptide sets and different proteins is shown in Table 2. The average magnitude of responses for each allele (total magnitude of responses detected for that allele corrected for the number of donors tested) was 2928 ± 2485.9 IFNγ SFCs/10^6^ PBMCs, and the average number of epitopes recognized per donor in the context of each allele was 5 ± 3 epitopes/donor. In particular, certain alleles, such as DRB1*16:02, DRB1*13:01, DRB1*11:04, and DRB1*01:01 were associated with a relatively high magnitude of response (>4600 SFCs), while DRB1*10:01, DRB1*15:02, DRB1*08:02, DRB1*04:07, and DRB1*04:01 were associated a moderate magnitude of response (>2280 SFCs). Other alleles, such as DRB1*04:03, DRB1*15:01, DRB1*11:01, DRB1*07:01, DRB1*03:01, DRB1*15:06, and DRB1*12:01 were associated with relatively weak magnitudes of response (<2280 SFCs) and accounted for less than 17% of the total response when combined. 

### 3.5. Development and Validation of a CD4^+^ Yellow Fever Epitope Mega Pool

**Figure 2:**
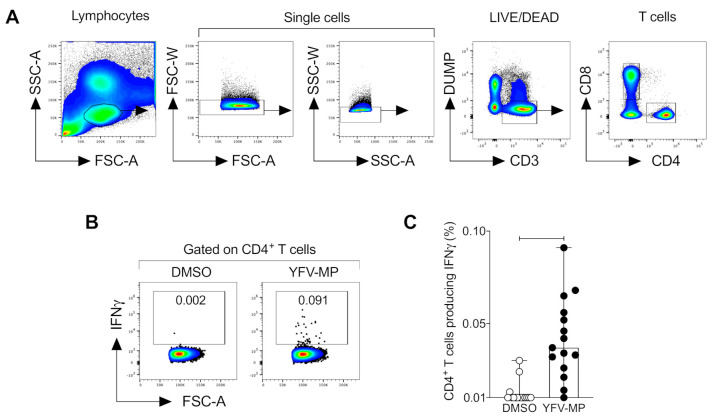
Validation of YVF-MP in vaccine individuals. (**A**) Representative dot plot of the gating strategy for CD4^+^ T cell selection. Lymphocytes were identified in forward scatter (FSC), and side scatter (SSC) plots. The doublets, CD14^+^, and CD19^+^ cells were excluded. Next, gated on CD3^+^ cells, the CD4^+^ and CD8^+^ population was selected. (**B**) Representative dot plot of gating strategy for antigen-specific CD4^+^ T cells producing IFNγ after DMSO or YFV-MP stimulation in the same donor. Fifteen samples from YF-17D vaccinees were used for YFV-MP validation. (**C**) Comparison of percentage of CD4^+^ T cells producing IFNγ between DMSO and YFV-MP stimulated cells. Data are expressed as the median and the range. Statistical analyses were performed using a two-tailed Wilcoxon test. *p* = 0.001.

To enable the detection of ex vivo CD4^+^ T cell response elicited by the YF-17D vaccine, we applied a three-fold approach to develop a YFV mega pool (YFV-MP) as outlined in more detail in material and methods. Next, we validate the YFV CD4 MP measuring the IFNγ production of antigen-specific CD4^+^ T cells (Figure 2A,B). A greater percentage of antigen-specific CD4^+^ T cells producing IFNγ after YFV-MP stimulation was observed than in DMSO cultured cells (Figure 2C). 

## 4. Discussion

YFV is a mosquito-borne member of the genus flavivirus, including other important human-pathogenic viruses genetically related, such as dengue, Japanese encephalitis, West Nile, and Zika viruses. Out of all flavivirus, a licensed vaccine is only available against DENV, JEV, and YFV, although they present with different efficacy profiles. For example, a safety signal in dengue seronegative vaccine recipients has sparked some concern on the widespread application of Dengvaxia [28], while vaccines against JEV and YFV have been administered successfully in humans for decades [44]. The vaccine against YFV has proven to be amongst the most successful vaccines in terms of risk factors and the induction of long-term protection for at least 10 years [45,46]. However, a current resurgence of YF in the metropolitan areas of Brazil has sparked renewed interest in the exact phenotypes found in the general population. We have previously shown in the case of natural infection with DENV that a variation in terms of frequency of response and magnitude exists [47]. It is thus important to comprehensively address the CD4^+^ T cell response covering the most frequently expressed HLA phenotypes in a given population. We analyzed the class II epitopes restricted by HLA-DRB1 alleles, a locus that worldwide has been observed to be associated with stronger responses to flavivirus epitopes, compared to other HLA class II loci [48]. Here, it was found that a high-to-moderate fraction of the response, in terms of magnitude, was restricted by HLA-DRB1*16:02, HLA-DRB1*13:01, HLA-DRB1*11:04, HLA-DRB1*01:01, DRB1*10:01, DRB1*15:02, DRB1*08:02, DRB1*04:07, and DRB1*04:01 alleles in YFV vaccinated individuals from the San Diego area. Interestingly, these findings are related to the previous report showing that the DRB1*01:01, DRB1*08:02, and DRB1*04:01 alleles recognize YFV epitopes in a cohort of YFV vaccinated donors from Brazil [9]. Mechanism of vaccine protection [49]. While neutralizing antibodies against YFV have been widely recognized as the correlate of protection against re-infection, the importance of cellular immunity has been acknowledged [44]. Particularly, CD4^+^ T cells have been shown to contribute by providing help to both CD8^+^ T cells and antibody-producing B cells [50]. 

CD4 epitopes derived from YF-17D have previously been reported [7,8,9,10,11,12,13,14,15,16,17,18,19]. However, most studies have focused on a limited amount of HLA alleles that failed to address the diversity of HLA.

Identifying immunodominant epitopes in YFV vaccinees is fundamental for the characterization of the adaptive immune response against YF [51,52]. In the present study, CD4^+^ T cells from YFV vaccinated donors recognized both structural and non-structural proteins and preferably targets the C, E, NS3, and NS5 proteins. Although few studies have identified YFV class II epitopes, a previous report found a set of 108 immunogenic YFV peptides from the envelope and non-structural proteins in YF vaccinated donors from Brazil (ten out of those peptides that belong to E, NS2A, NS2B, NS3, and NS4A proteins, were included in our study) and showed that a high number of NS3 and NS5 peptides were recognized by T cell from vaccinated donors, which is consistent with our findings [9]. In addition, a previous report identified a strong CD4^+^ T cell response to epitopes derived from the C and E proteins of YFV in a cohort of YFV vaccinated individuals [19]. Similarly, previous studies in other flavivirus models, like Dengue and Zika, have been shown that the CD4^+^ T cell response targets to C, E, NS3, and NS5 [34,35,51], suggesting that the structural proteins from capsid and envelope, as well as the non-structural proteins NS3 and NS5, are highly immunodominant, a feature that is shared between the flaviviruses [2]. In a parallel study, we have investigated the potential cross-reactivity of some of the epitopes identified and found limited cross-reactivity of epitopes induced by YF17D vaccination with other flaviviruses, such as DENV, ZIKV, and JEV [53]. The knowledge of immunodominance is highly relevant in the context of vaccine design, particularly as chimeric flavivirus vaccines, have been designed. Excluding proteins that are dominant T cell targets might have a direct influence on the efficacy of such vaccine constructs.

## 5. Conclusions

Comprehensive epitope mapping across all ten yellow fever proteins revealed patterns of immunodominance of YFV-specific CD4^+^ T cell responses that preferably target the C, E, NS3, and NS5 proteins. The subsequent development of a CD4 YFV-specific MP allows the ex vivo identification of YFV-specific responses in small amounts of blood samples, irrespective of the HLA phenotype, which provides a valuable tool in the evaluation of vaccine-specific CD4^+^ T cells. 

## Figures and Tables

**Table 1:** Most dominant peptides are recognized by YF-17D vaccinees. PBMCs, peripheral blood mononuclear cells.

Sequence	Protein	Position	Total SFC (10^6^ PBMCs)	Cumulative % Total	HLA Recognized	HLA Restriction(s)
VRRGVRSLSNKIKQK	C	16	7333	5.4	4	DRB1*04:03, DRB1*08:02, DRB1*15:01, DRB1*16:02
LMRRMRRPTGKVTLE	NS5	2746	6330	10.0	4	DRB1*11:01, DRB1*11:04, DRB1*13:01, DRB1*15:02
QTSRLLMRRMRRPTG	NS5	2741	5313	13.8	5	DRB1*08:02, DRB1*11:01, DRB1*11:04, DRB1*13:01, DRB1*15:02
ILAECARRRLRTLVL	NS3	1696	4983	17.5	1	DRB1*16:02
VVVLNRKTFEREYPT	NS3	1871	4873	21.0	2	DRB1*11:04, DRB1*13:01
LRKVKRVVASLMRGL	C	81	4587	24.4	4	DRB1*01:01, DRB1*04:03, DRB1*11:01, DRB1*11:04
GEVIGLYGNGILVGD	NS3	1631	3757	27.1	2	DRB1*15:01, DRB1*15:02
RFLPQILAECARRRL	NS3	1691	3480	29.7	2	DRB1*04:07, DRB1*10:01
QGLAVLRKVKRVVAS	C	76	2893	31.8	4	DRB1*08:02, DRB1*11:01, DRB1*11:04, DRB1*13:01
DMRLLSLAVSSAVPT	NS5	3281	2793	33.8	4	DRB1*04:07, DRB1*07:01, DRB1*11:01, DRB1*13:01
KAGKSVVVLNRKTFE	NS3	1866	2773	35.8	2	DRB1*11:04, DRB1*13:01
GVTLVRKNRWLLLNV	M	121	2693	37.8	3	DRB1*08:02, DRB1*13:01, DRB1*15:01
SLASVAMCRTPFSLA	NS4B	2436	2530	39.7	4	DRB1*04:03, DRB1*04:07, DRB1*10:01, DRB1*11:04
NNGGDAMYMALIAAF	NS2A	1196	2523	41.5	1	DRB1*13:01
ITAHLKRLWKMLDPR	C	61	2400	43.2	2	DRB1*11:01, DRB1*11:04
VRKVCYNAVLTHVKI	E	341	2393	45.0	3	DRB1*04:07, DRB1*10:01, DRB1*11:04
GSIVACAKFTCAKSM	E	396	2353	46.7	2	DRB1*11:04, DRB1*13:01
NNLYKLHGGHVSCRV	E	556	2287	48.4	1	DRB1*01:01
LLIGFGLRTLWSPRE	NS2A	1216	2233	50.0	2	DRB1*13:01, DRB1*15:01
GFIFFFLFNILTGKK	C	46	2227	51.6	5	DRB1*01:01, DRB1*04:01, DRB1*07:01, DRB1*11:01, DRB1*15:02
VVVQDPKNVYQRGTH	NS1	866	2123	53.2	2	DRB1*03:01, DRB1*13:01
RVVASLMRGLSSRKR	C	86	2087	54.7	2	DRB1*04:03, DRB1*13:01
GNTSLLWNGPMAVSM	NS4B	2466	2027	56.2	1	DRB1*01:01
KIERWFVRNPFFAVT	M	241	1960	57.6	2	DRB1*15:02, DRB1*16:02
EVDISVVVQDPKNVY	NS1	861	1957	59.0	1	DRB1*13:01
FVGVMYNLWKMKTGR	NS4B	2491	1900	60.4	2	DRB1*11:01, DRB1*11:04
LIWVGINTRNMTMSM	E	746	1790	61.7	3	DRB1*04:01, DRB1*10:01, DRB1*13:01
GTRKIMKVVNRWLFR	NS5	2876	1720	63.0	1	DRB1*15:01
DGDSYYYSEPTSENN	NS3	1956	1610	64.2	1	DRB1*04:01
PKNVYQRGTHPFSRI	NS1	871	1600	65.3	1	DRB1*16:02
FLFNILTGKKITAHL	C	51	1573	66.5	2	DRB1*01:01, DRB1*11:01
DEQEILNYMSPHHKK	NS5	3056	1513	67.6	3	DRB1*04:01, DRB1*15:01, DRB1*15:06
LPSIRAANVMAASLR	NS3	1851	1437	68.6	5	DRB1*04:01, DRB1*04:03, DRB1*04:07, DRB1*07:01, DRB1*11:04
GAFLVRNGKKLIPSW	NS3	1541	1413	69.7	3	DRB1*11:01, DRB1*13:01, DRB1*16:02
GVIMMFLSLGVGADQ	E	766	1313	70.6	2	DRB1*11:01, DRB1*15:01
QYVIRAQLHVGAKQE	E	421	1270	71.5	1	DRB1*16:02
VSMMIAMEVVLRKRQ	NS2A	1141	1243	72.5	3	DRB1*04:03, DRB1*13:01, DRB1*16:02
HATLTYRMLEPTRVV	NS3	1751	1213	73.3	2	DRB1*01:01, DRB1*10:01
RWLFRHLAREKNPRL	NS5	2886	1140	74.2	1	DRB1*13:01
FIAKVRSHAAIGAYL	NS5	2906	1127	75.0	3	DRB1*01:01, DRB1*04:03, DRB1*04:07

**Table 2:** CD4+ T cell immunoreactivity against YFV epitopes in PBMCs from YF-17D vaccinees.

HLA DRB1 Allele	No. ^1^	Total Response Magnitude/Donor	Average of Epitopes/Donor	C	M	E	NS1	NS2A	NS2B	NS3	NS4A	NS4B	NS5
01:01	3	4644	8.3	820	140	876	0	300	171	831	0	988	519
03:01	2	850	4.5	0	0	93	200	0	0	413	0	47	97
04:01	5	2280	4.6	179	0	297	0	0	64	1211	116	213	199
04:03	3	2093	3.3	1307	0	38	0	160	0	98	0	322	169
04:05	1	0	0.0	0	0	0	0	0	0	0	0	0	0
04:07	3	2577	4.3	0	0	647	0	51	0	1080	164	351	283
07:01	3	984	1.7	29	0	76	0	0	0	180	0	0	700
08:02	1	2993	6.0	1320	87	0	0	0	0	320	0	0	1267
10:01	1	4560	13.0	0	0	833	0	300	0	2253	0	793	380
11:01	4	1642	4.8	180	0	313	0	0	58	125	38	73	854
11:04	3	5185	5.8	1625	0	747	0	0	0	493	0	672	1648
12:01	2	577	1.5	0	0	70	0	0	0	80	0	0	427
13:01	5	5746	5.6	889	453	314	784	1023	0	1604	0	0	678
15:01	8	1767	2.1	391	72	93	0	240	0	556	108	16	292
15:02	2	3105	6.5	57	893	0	0	0	0	182	237	182	1555
15:06	1	820	2.0	0	0	0	0	0	0	0	0	250	570
16:02	1	9950	10.0	573	320	1450	1600	290	0	5097	0	140	480
TOTAL	48	49,773	-	7369	1965	5846	2584	2365	293	14,523	662	4047	10,117
%	-	100	-	14.8	3.9	11.7	5.2	4.8	0.6	29.2	1.3	8.1	20.3
Mean	-	2928	5	433	116	344	152	139	17	854	39	238	595
SD	-	2485.9	3.3	553.9	238.6	421.6	420.0	258.4	44.5	1261.6	72.3	305.0	486.7

^1^ Number of HLA tested in the donor cohort included in the epitope identification studies.

## Supplementary Materials

The following are available online at https://www.mdpi.com/1999-4915/12/11/1300/s1, Figure S1. Frequency of HLA-DRB1* alleles in the donor cohort, Table S1. List of donors for epitope identification studies, Table S2. List of donors for YFV-CD4 MP validation, Table S3. Epitopes included in the CD4 YFV-MP, Table S4. List of all YFV peptides identified.

## References

[1] WHO (2019). Yellow fever in Africa and the Americas, 2018. Wkly. Epidemiol. Rec..

[2] Hasan S.S., Sevvana M., Kuhn R.J., Rossmann M.G. (2018). Structural biology of Zika virus and other flaviviruses. Nat. Struct. Mol. Biol..

[3] Douam F., Ploss A. (2018). Yellow fever virus: Knowledge gaps impeding the fight against an old foe. Trends Microbiol..

[4] CDC Yellow Fever Vaccine. https://www.cdc.gov/yellowfever/vaccine/index.html.

[5] Ferreira C.C., Campi-Azevedo A.C., Peruhype-Magalhaes V., Costa-Pereira C., Albuquerque C.P., Muniz L.F., Yokoy de Souza T., Oliveira A.C.V., Martins-Filho O.A., da Mota L.M.H. (2018). The 17D-204 and 17DD yellow fever vaccines: An overview of major similarities and subtle differences. Expert Rev. Vaccines.

[6] Poland J.D., Calisher C.H., Monath T.P., Downs W.G., Murphy K. (1981). Persistence of neutralizing antibody 30-35 years after immunization with 17D yellow fever vaccine. Bull. World Health Organ..

[7] Co M.D., Terajima M., Cruz J., Ennis F.A., Rothman A.L. (2002). Human cytotoxic T lymphocyte responses to live attenuated 17D yellow fever vaccine: Identification of HLA-B35-restricted CTL epitopes on nonstructural proteins NS1, NS2b, NS3, and the structural protein E. Virology.

[8] Guy B., Nougarede N., Begue S., Sanchez V., Souag N., Carre M., Chambonneau L., Morrisson D.N., Shaw D., Qiao M. (2008). Cell-mediated immunity induced by chimeric tetravalent dengue vaccine in naive or flavivirus-primed subjects. Vaccine.

[9] de Melo A.B., Nascimento E.J., Braga-Neto U., Dhalia R., Silva A.M., Oelke M., Schneck J.P., Sidney J., Sette A., Montenegro S.M. (2013). T-cell memory responses elicited by yellow fever vaccine are targeted to overlapping epitopes containing multiple HLA-I and -II binding motifs. PLoS Negl. Trop. Dis..

[10] Blom K., Braun M., Ivarsson M.A., Gonzalez V.D., Falconer K., Moll M., Ljunggren H.G., Michaelsson J., Sandberg J.K. (2013). Temporal dynamics of the primary human T cell response to yellow fever virus 17D as it matures from an effector- to a memory-type response. J. Immunol..

[11] Wieten R.W., Jonker E.F., van Leeuwen E.M., Remmerswaal E.B., Ten Berge I.J., de Visser A.W., van Genderen P.J., Goorhuis A., Visser L.G., Grobusch M.P. (2016). A single 17D yellow fever vaccination provides lifelong immunity; characterization of yellow fever-specific neutralizing antibody and T-cell responses after vaccination. PLoS ONE.

[12] Wieten R.W., Goorhuis A., Jonker E.F.F., de Bree G.J., de Visser A.W., van Genderen P.J.J., Remmerswaal E.B.M., Ten Berge I.J.M., Visser L.G., Grobusch M.P. (2016). 17D yellow fever vaccine elicits comparable long-term immune responses in healthy individuals and immune-compromised patients. J. Infect..

[13] Lee E.S., Thomas P.G., Mold J.E., Yates A.J. (2017). Identifying T cell receptors from high-throughput sequencing: Dealing with promiscuity in TCRalpha and TCRbeta pairing. PLoS Comput. Biol..

[14] Pogorelyy M.V., Minervina A.A., Touzel M.P., Sycheva A.L., Komech E.A., Kovalenko E.I., Karganova G.G., Egorov E.S., Komkov A.Y., Chudakov D.M. (2018). Precise tracking of vaccine-responding T cell clones reveals convergent and personalized response in identical twins. Proc. Natl. Acad. Sci. USA.

[15] Akondy R.S., Johnson P.L., Nakaya H.I., Edupuganti S., Mulligan M.J., Lawson B., Miller J.D., Pulendran B., Antia R., Ahmed R. (2015). Initial viral load determines the magnitude of the human CD8 T cell response to yellow fever vaccination. Proc. Natl. Acad. Sci. USA.

[16] Akondy R.S., Monson N.D., Miller J.D., Edupuganti S., Teuwen D., Wu H., Quyyumi F., Garg S., Altman J.D., Del Rio C. (2009). The yellow fever virus vaccine induces a broad and polyfunctional human memory CD8+ T cell response. J. Immunol..

[17] Kongsgaard M., Bassi M.R., Rasmussen M., Skjodt K., Thybo S., Gabriel M., Hansen M.B., Christensen J.P., Thomsen A.R., Buus S. (2017). Adaptive immune responses to booster vaccination against yellow fever virus are much reduced compared to those after primary vaccination. Sci. Rep..

[18] Wrammert J., Miller J., Akondy R., Ahmed R. (2009). Human immune memory to yellow fever and smallpox vaccination. J. Clin. Immunol..

[19] Koblischke M., Mackroth M.S., Schwaiger J., Fae I., Fischer G., Stiasny K., Heinz F.X., Aberle J.H. (2017). Protein structure shapes immunodominance in the CD4 T cell response to yellow fever vaccination. Sci. Rep..

[20] da Costa-Rocha I.A., Campi-Azevedo A.C., Peruhype-Magalhaes V., Coelho-Dos-Reis J.G., Fradico J.R.B., Souza-Lopes T., Reis L.R., Freire L.C., Costa-Pereira C., Mambrini J.V.M. (2019). Duration of humoral and cellular immunity 8 years after administration of reduced doses of the 17DD-yellow fever vaccine. Front. Immunol..

[21] Domingo C., Fraissinet J., Ansah P.O., Kelly C., Bhat N., Sow S.O., Mejia J.E. (2019). Long-term immunity against yellow fever in children vaccinated during infancy: A longitudinal cohort study. Lancet Infect. Dis..

[22] Akondy R.S., Fitch M., Edupuganti S., Yang S., Kissick H.T., Li K.W., Youngblood B.A., Abdelsamed H.A., McGuire D.J., Cohen K.W. (2017). Origin and differentiation of human memory CD8 T cells after vaccination. Nature.

[23] Tian Y., Grifoni A., Sette A., Weiskopf D. (2019). Human T cell response to dengue virus infection. Front. Immunol..

[24] Slon Campos J.L., Mongkolsapaya J., Screaton G.R. (2018). The immune response against flaviviruses. Nat. Immunol..

[25] Waggoner J.J., Rojas A., Pinsky B.A. (2018). Yellow fever virus: Diagnostics for a persistent arboviral threat. J. Clin. Microbiol..

[26] Ndeffo-Mbah M.L., Pandey A. (2019). Global risk and elimination of yellow fever epidemics. J. Infect. Dis..

[27] Zhao S., Stone L., Gao D., He D. (2018). Modelling the large-scale yellow fever outbreak in Luanda, Angola, and the impact of vaccination. PLoS Negl. Trop. Dis..

[28] Thomas S.J., Yoon I.K. (2019). A review of Dengvaxia(R): Development to deployment. Hum. Vaccin. Immunother..

[29] Pham J., Oseroff C., Hinz D., Sidney J., Paul S., Greenbaum J., Vita R., Phillips E., Mallal S., Peters B. (2016). Sequence conservation predicts T cell reactivity against ragweed allergens. Clin. Exp. Allergy.

[30] Vita R., Mahajan S., Overton J.A., Dhanda S.K., Martini S., Cantrell J.R., Wheeler D.K., Sette A., Peters B. (2019). The Immune Epitope Database (IEDB): 2018 update. Nucleic Acids Res..

[31] Kim Y., Ponomarenko J., Zhu Z., Tamang D., Wang P., Greenbaum J., Lundegaard C., Sette A., Lund O., Bourne P.E. (2012). Immune epitope database analysis resource. Nucleic Acids Res..

[32] Wang P., Sidney J., Dow C., Mothe B., Sette A., Peters B. (2008). A systematic assessment of MHC class II peptide binding predictions and evaluation of a consensus approach. PLoS Comput. Biol..

[33] Wang P., Sidney J., Kim Y., Sette A., Lund O., Nielsen M., Peters B. (2010). Peptide binding predictions for HLA DR, DP and DQ molecules. BMC Bioinformatics.

[34] Weiskopf D., Angelo M.A., Grifoni A., O’Rourke P.H., Sidney J., Paul S., De Silva A.D., Phillips E., Mallal S., Premawansa S. (2016). HLA-DRB1 alleles are associated with different magnitudes of dengue virus-specific CD4+ T-cell responses. J. Infect. Dis..

[35] Grifoni A., Angelo M.A., Lopez B., O’Rourke P.H., Sidney J., Cerpas C., Balmaseda A., Silveira C.G.T., Maestri A., Costa P.R. (2017). Global assessment of dengue virus-specific CD4(+) T cell responses in dengue-endemic areas. Front. Immunol..

[36] Sidney J., Peters B., Frahm N., Brander C., Sette A. (2008). HLA class I supertypes: A revised and updated classification. BMC Immunol..

[37] Weiskopf D., Yauch L.E., Angelo M.A., John D.V., Greenbaum J.A., Sidney J., Kolla R.V., De Silva A.D., de Silva A.M., Grey H. (2011). Insights into HLA-restricted T cell responses in a novel mouse model of dengue virus infection point toward new implications for vaccine design. J. Immunol..

[38] Carrasco Pro S., Sidney J., Paul S., Lindestam Arlehamn C., Weiskopf D., Peters B., Sette A. (2015). Automatic generation of validated specific epitope sets. J. Immunol. Res..

[39] Bonaldo M.C., Gomez M.M., Dos Santos A.A., Abreu F.V.S., Ferreira-de-Brito A., Miranda R.M., Castro M.G., Lourenco-de-Oliveira R. (2017). Genome analysis of yellow fever virus of the ongoing outbreak in Brazil reveals polymorphisms. Mem. Inst. Oswaldo Cruz.

[40] Paul S., Lindestam Arlehamn C.S., Scriba T.J., Dillon M.B., Oseroff C., Hinz D., McKinney D.M., Carrasco Pro S., Sidney J., Peters B. (2015). Development and validation of a broad scheme for prediction of HLA class II restricted T cell epitopes. J. Immunol. Methods.

[41] Paul S., Sidney J., Sette A., Peters B. (2016). TepiTool: A pipeline for computational prediction of T cell epitope candidates. Curr. Protoc. Immunol..

[42] Dhanda S.K., Vaughan K., Schulten V., Grifoni A., Weiskopf D., Sidney J., Peters B., Sette A. (2018). Development of a novel clustering tool for linear peptide sequences. Immunology.

[43] Greenbaum J., Sidney J., Chung J., Brander C., Peters B., Sette A. (2011). Functional classification of class II human leukocyte antigen (HLA) molecules reveals seven different supertypes and a surprising degree of repertoire sharing across supertypes. Immunogenetics.

[44] Liang H., Lee M., Jin X. (2016). Guiding dengue vaccine development using knowledge gained from the success of the yellow fever vaccine. Cell. Mol. Immunol..

[45] Monath T.P. (2012). Review of the risks and benefits of yellow fever vaccination including some new analyses. Expert Rev. Vaccines.

[46] Roukens A.H.E., van Halem K., de Visser A.W., Visser L.G. (2018). Long-term protection after fractional-dose yellow fever vaccination: Follow-up study of a randomized, controlled, noninferiority trial. Ann. Intern. Med..

[47] Grifoni A., Moore E., Voic H., Sidney J., Phillips E., Jadi R., Mallal S., De Silva A.D., De Silva A.M., Peters B. (2019). Characterization of magnitude and antigen specificity of HLA-DP, DQ, and DRB3/4/5 restricted DENV-specific CD4+ T cell responses. Front. Immunol..

[48] Grifoni A., Weiskopf D., Lindestam Arlehamn C.S., Angelo M., Leary S., Sidney J., Frazier A., Phillips E., Mallal S., Mack S.J. (2018). Sequence-based HLA-A, B, C, DP, DQ, and DR typing of 714 adults from Colombo, Sri Lanka. Hum. Immunol..

[49] Kallas E.G., Wilder-Smith A. (2019). Managing severe yellow fever in the intensive care: Lessons learnt from Brazil. J. Travel Med..

[50] Barrett A.D., Teuwen D.E. (2009). Yellow fever vaccine-how does it work and why do rare cases of serious adverse events take place?. Curr. Opin. Immunol..

[51] Grifoni A., Pham J., Sidney J., O’Rourke P.H., Paul S., Peters B., Martini S.R., de Silva A.D., Ricciardi M.J., Magnani D.M. (2017). Prior dengue virus exposure shapes T cell immunity to Zika virus in humans. J. Virol..

[52] Lim M.Q., Kumaran E.A.P., Tan H.C., Lye D.C., Leo Y.S., Ooi E.E., MacAry P.A., Bertoletti A., Rivino L. (2018). Cross-reactivity and anti-viral function of dengue capsid and NS3-specific memory T cells toward Zika virus. Front. Immunol..

[53] Grifoni A., Voic H., Dhanda S.K., Kidd C.K., Brien J.D., Buus S., Stryhn A., Durbin A.P., Whitehead S., Diehl S.A. (2020). T cell responses induced by attenuated flavivirus vaccination are specific and show limited cross-reactivity with other Flavivirus species. J. Virol..

