# Supplementary material for: Identification of Novel Yellow Fever Class II Epitopes in YF-17D Vaccinees
Source: Viruses. 2020 Nov 12;12(11):1300. doi: 10.3390/v12111300 (PMC7697718; doi:10.3390/v12111300)
Supplement: Supplementary file 1 [file viruses-12-01300-s001.pdf]

viruses

Article

Identification of Novel Yellow Fever Class II Epitopes in YF-17D Vaccinees

Jose Mateus [1], Alba Grifoni [1], Hannah Voic [1], Michael A. Angelo [1], Elizabeth Phillips [2], Simon Mallal [2], John Sidney [1], Alessandro Sette [1,3,*] and Daniela Weiskopf [1,*]

[1] Center for Infectious Disease and Vaccine Research, La Jolla Institute for Immunology (LJI), La Jolla, CA 92037, USA; jmtrivino@lji.org (J.M.); agrifoni@lji.org (A.G.); hvoic@lji.org (H.V.); mikey.angelo88@gmail.com (M.A.A.); jsidney@lji.org (J.S.)

[2] Institute for Immunology and Infectious Diseases, Murdoch University, Perth, WA 6150, Australia; E.Phillips@murdoch.edu.au (E.P.); S.Mallal@murdoch.edu.au (S.M.)

3 Department of Medicine, Division of Infectious Diseases and Global Public Health, University of California, San Diego (UCSD), La Jolla, CA 92037, USA

* Correspondence: alex@lji.org (A.S.); dweiskopf@lji.org (D.W.); Tel.: +1-85-8752-6919 (A.S. & D.W.)

Supplementary materials

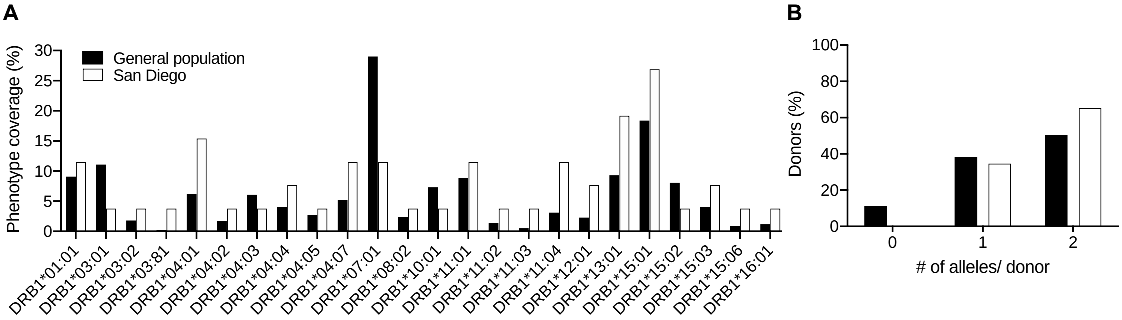

Figure S1. Frequency of HLA-DRB1* alleles in the donor cohort. (**A**) Frequency of HLA-DRB1* alleles in the donor cohort. The phenotypic frequency of HLA-DRB1* alleles in the donor cohort studied here is plot, and shown in comparison with corresponding allele frequency in a collection of over 3500 donor samples from various clinic sites around the world, to include the Americas, Asia, South Africa and Europe. Typing was performed as described in the Methods. (**B**) HLA-DRB1 prediction panel provides broad coverage. A panel of 17 HLA-DRB1 alleles was selected to predict potential YFV epitopes, as described in the Methods. The plot shows the percent of individuals in the study cohort (dark bars) and the general population (light bars) that have allele matches for zero, one or both DRB1 haplotypes.

Table S1. List of donors for epitope identification studies.

Donor ID	Gender	Age (years)	Months post-vaccination	HLA-DRB1 alleles	
1290	Male	36	122	12:01	15:06
2026	Male	41	48	11:04	15:03
2027	Female	25	59	11:04	13:01
2028	Female	35	21	13:01	15:01
2029	Female	27	84	04:01	15:03
2031	Male	44	7	04:03	07:01
2032	Male	21	120	04:02	11:01
2033	Female	34	60	04:01	11:04
2034	Male	18	115	07:01	13:01
2035	Male	35	144	13:01	16:01
2078	Male	26	101	11:03	15:01
2222	Female	42	112	03:01	03:81
2223	Female	33	24	12:01	12:01
2224	Female	18	115	01:01	11:01
2252	Male	55	115	04:05	07:01
2257	Male	42	24	04:04	15:01
2262	Male	25	73	04:07	15:01
2263	Male	23	32	04:01	15:02
2264	Female	48	61	10:01	15:01
2265	Male	54	61	03:02	04:07
2374	Female	26	50	01:01	13:01
2388	Female	44	108	04:04	11:01
2392	Male	29	85	01:01	01:01
2395	Male	33	106	04:01	15:01
2425	Male	59	32	08:02	11:02
2452	Female	22	42	04:07	15:01

Table S2. List of donors for YFV-CD4 MP validation

Donor ID	Gender	Age (years)
1711	Female	20
2223	Female	33
2227	Male	18
2267	Male	55
2389	Female	20
2421	Female	24
2454	Female	46
2550	Female	60
2584	Female	32
2701	Male	25
2733	Male	31
2928	Male	20
3111	Female	59
3147	Female	34
3156	Male	27

Table S3. Epitopes included in the CD4 YFV-MP

Sequence	Protein	Start	End
LGVNMVRRGVRSLSN	C	11	25
VRRGVRSLSNKIKQK	C	16	30
NKIKQKTKQIGNRPGPS	C	25	41
GFIFFFLFNILTGKKITAHL	C	46	65
FFFLFNILTGKKITAHL	C	49	65
LTGKKITAHLKRLWK	C	56	70
ITAHLKRLWKMLDPR	C	61	75
KRLWKMLDPRQGLAVLRK	C	66	83
DPRQGLAVLRKVKRVVASL	C	73	91
QGLAVLRKVKRVVASLMRGL	C	76	95
GLAVLRKVKRVVASLMRGL	C	77	95
AVLRKVKRVVASLMR	C	79	93
KRVVASLMRGLSSRK	C	85	99
LRKVKRVVASLMRGL	C	81	95
VVASLMRGLSSRKRR	C	87	101
KRVVASLMRGLSSRK	C	85	99
LMRGLSSRKRRSHDV	C	91	105
KRVVASLMRGLSSRKRR	C	85	101
RSQDVLTIQFLILGM	C	101	115
LTVQFLILGMLLMTG	C	106	120
LILGMLLMTGGVTLV	pr	111	125
GVTLVRKNRWLLLNV	pr	121	135
RKNRWLLLNVTSEDL	pr	126	140
IDCWCYGVENVRVAYGK	pr	182	198
ERQLQKIERWFVRNP	M	236	250
KIERWFVRNPFFAVTALT	M	241	258
IERWFVRNPFFAVTALTIA	M	242	260
LVRNPFFAVTALTIA	M	246	260
FFAVTALTIAYLVGSNMTQR	M	251	270
FFAVTALTIAYLVGS	M	251	265
VTALTIAYLVGSNMTQR	M	254	270
NMTQRVVIALLVLAV	M	266	280
VVIALLVLAVGPAYS	M	271	285
LVLAVGPAYSAHCIG	M	276	290
ISLETVAIDRPAEVRKV	E	328	344
ARKVCYNAVLTHVKI	E	341	355
GKGSIVACAKFTCAKSM	E	394	410
GSIVACAKFTCAKSM	E	396	410
AKFTCAKSMSLFEVD	E	398	412
CAKSMSLFEVDQTKIQYVIR	E	406	425
SLFEVDQTKIQYVIRAQL	E	411	428
LFEVDQTKIQYVIRAQLHV	E	412	430
QYVIRAQLHVGAKQE	E	421	435
KTLKFDALSGSQEVEFI	E	442	458
ETESWIVDRQWAQDLTL	E	484	500
ESWIVDRQWAQDLTL	E	486	500
RQWAQDLTLPWQSGS	E	492	506

SGGVWREMHHLVEFEPPHA	E	506	524
PPHAATIRVLALGNQ	E	521	535
TIRVLALGNQEGSLKTA	E	526	542
EGSLKTALTGAMRVT	E	536	550
NNLYKLHGGHVSCRVKL	E	556	572
VSCRVKLSALTLKGTSYKI	E	566	584
CRVKLSALTLKGTSYKIC	E	568	585
SYKICTDKMFFVKNP	E	581	595
TDKMFFVKNPTDTGHGT	E	586	602
TGHGTVVMQVKVSKGAPC	E	598	615
RIPVIVADDLTAAINKGILV	E	616	635
RIPVIVADDLTAAINKG	E	616	632
AINKGILVTVNPIASTND	E	628	645
STNDDEVLIEVNPPFGDSY	E	642	660
DSYIIVGRGDSRLTYQW	E	658	674
LTYQWHKEGSSIGKLFT	E	670	686
DTAWDFSSAGGFFTS	E	700	714
SSAGGFFTSVGKGIHTVFGS	E	706	725
SSAGGFFTSVGKGIHTV	E	706	722
TVFGSAFQGLFGGLS	E	721	735
FGGLSWITKVIMGAV	E	731	745
WITKVIMGAVLIWVG	E	736	750
MGAVLIWVGINTRNMTMSM	E	742	760
INTRNMTMSMSMILV	E	751	765
MTMSMSMILVGVIMM	E	756	770
SMILVGVIMMFLSLG	E	761	775
GVIMMFLSLGVGADQ	E	766	780
NFGKRELKCGDGIFIFR	NS1	785	801
KYSYYPEDPVKLASIVK	NS1	809	825
DPVKLASIVKASFEE	NS1	816	830
CGLNSVDSLEHEMWRSR	NS1	833	849
DSLEHEMWRSRADEINA	NS1	839	855
EVDISVVVQDPKNVYQRGT	NS1	861	879
DISVVVQDPKNVYQRGTH	NS1	863	880
PKNVYQRGTHPFSRI	NS1	871	885
DGLQYGWKTWGKNLVFS	NS1	887	903
WGKNLVFSPGRKNGS	NS1	896	910
GRKNGSFIIDGKSRKEC	NS1	905	921
NSFQIEEFGTGVFTTRV	NS1	929	945
FTTRVYMDAVFEYTIDC	NS1	941	957
YTIDCDGSILGAAVNGK	NS1	953	969
NGTWMIHTLEALDYK	NS1	986	1000
ECEWPLTHTIGTSVEES	NS1	1001	1017
VPFGLVSMMIAMEVV	NS2A	1136	1150
VSMMIAMEVVLRKRQGPKQ	NS2A	1141	1159
VLLGAMLVGQVTLLD	NS2A	1166	1180
MLVGQVTILDLLKLT	NS2A	1171	1185
VTLLDLLKLTVAVGLHFHEM	NS2A	1176	1195
LDLLKLTVAVGLHFHEM	NS2A	1179	1195
NNGGDAMYMALIAAFSIR	NS2A	1196	1213
NGGDAMYMALIAAFSIRPG	NS2A	1197	1215

LIAAFSIRPGLLIGF	NS2A	1206	1220
LLIGFGLRTLWSPRE	NS2A	1216	1230
GLRTLWSPRERLVLT	NS2A	1221	1235
LGGVMGGLWKYLNAV	NS2A	1245	1259
LNAVSLCILTINAVA	NS2A	1256	1270
LCILTINAVASRKAS	NS2A	1261	1275
SRKASNVILPLMALL	NS2A	1271	1285
SNTILPLMALLTPVTMA	NS2A	1275	1291
TPVTMAEVRLAAMFF	NS2A	1286	1300
AEVRLATMLFCTVVI	NS2A	1291	1305
CAVVIIGVLHQNFKD	NS2A	1301	1315
IGVLHQNFKDTSMQK	NS2A	1306	1320
TSMQKTIPLVALTLT	NS2A	1316	1330
TIPLVALTLTSYLGL	NS2A	1321	1335
ALTLTSYLGLTQPFL	NS2A	1326	1340
SYLGLTQPFLGLCAF	NS2A	1331	1345
TQPFLGLCAFLATRI	NS2A	1336	1350
GLCAFLATRIFGRRS	NS2A	1341	1355
GLVGVLAGLAFQEME	NS2B	1366	1380
LAGLAFQEMENFLGPIAVG	NS2B	1371	1389
IAVGGILMMLVSVAG	NS2B	1386	1400
LLMMLVSVAGRVDGL	NS2B	1391	1405
SEQGEFKLLSEEKVPWD	NS2B	1433	1449
VPWDQVVMTSLALVG	NS2B	1446	1460
VVMTSLALVGAALHP	NS2B	1451	1465
LALVGAALHPFALLL	NS2B	1456	1470
FALLLVLAGWLFHVR	NS2B	1466	1480
VLAGWLFHVRGARRS	NS2B	1471	1485
SGDVLWDIPTPKIIEEC	NS3	1485	1501
HLEDGIYGIFQSTFLGAS	NS3	1503	1520
VFHTMWHVTRGAFLVRNGK	NS3	1531	1549
GAFLVRNGKKLIPSW	NS3	1541	1555
RNGKKLIPSWASVKE	NS3	1546	1560
DLVAYGGSWKLDGRW	NS3	1561	1575
WDGEEEVQLIAAVPG	NS3	1575	1589
VQLIAAVPGKNVVNV	NS3	1581	1595
NVVNVQTKPSLFKVR	NS3	1591	1605
RNGEVIGLYGNGILVGD	NS3	1629	1645
GEVIGLYGNGILVGD	NS3	1631	1645
GLYGNGILVGDNSFVSA	NS3	1635	1651
ILVGDNSFVSAISQT	NS3	1641	1655
NSFVSAISQTEVKEE	NS3	1646	1660
QEIPTMLKKGMTTVL	NS3	1666	1680
AGKTRRFLPQILAEC	NS3	1686	1700
RFLPQILAECARRRL	NS3	1691	1705
ILAECARRRLRTLVL	NS3	1696	1710
ARRRLRTLVLAPTRV	NS3	1701	1715
RTLVLAPTRVVLSEM	NS3	1706	1720
APTRVVLSEMKEAFHGLDV	NS3	1711	1729
GLDVKFHTQAFSAHG	NS3	1726	1740
IDAMCHATLTYRMLEPTRVV	NS3	1746	1765

MCHATLTYRMLEPTRVV	NS3	1749	1765
YRMLEPTRVVNWEVI	NS3	1756	1770
PTRVVNWEVIIMDEA	NS3	1761	1775
NWEVIIMDEAHFLDPASI	NS3	1766	1783
NESATILMTATPPGT	NS3	1796	1810
WNTGHDWILADKRPTAWF	NS3	1833	1850
GHDWILADKRPTAWFLPSIR	NS3	1836	1855
RPTAWFLPSIRAANVMA	NS3	1845	1861
LPSIRAANVMAASLR	NS3	1851	1865
LPSIRAANVMAASLRKAGKS	NS3	1851	1870
LPSIRAANVMAASLRKA	NS3	1851	1867
AASLRKAGKSVVVLN	NS3	1861	1875
KAGKSVVVLNRKTFE	NS3	1866	1880
VVVLNRKTFEREYPT	NS3	1871	1885
REYPTIKQKKPDFIL	NS3	1881	1895
PDFILATDIAEMGANLCVE	NS3	1891	1909
GRKVAIKGPLRISAS	NS3	1926	1940
IKGPLRISASSAAQR	NS3	1931	1945
SYYYSEPTSENNAHHVC	NS3	1959	1975
AHHVCWLEASMLLDN	NS3	1971	1985
FRELVRNCDLPVWLS	NS3	2021	2035
PVWLSWQVAKAGLKT	NS3	2031	2045
LSEFIKFAEGRRGAA	NS3	2096	2110
EVLVVLSELPDFLAK	NS4A	2111	2125
EEGSRAYRNALSMMP	NS4A	2141	2155
AYRNALSMMPEAMTI	NS4A	2146	2160
EAMTIVMLFILAGLL	NS4A	2156	2170
VMLFILAGLLTSGMV	NS4A	2161	2175
LAGLLTSGMVIFFMS	NS4A	2166	2180
TSGMVIFFMSPKGISRMSMA	NS4A	2171	2190
MVIFFMSPKGISRMSMA	NS4A	2174	2190
PKGISRMSMAMGTMA	NS4A	2181	2195
GCGYLMFLGGVKPTH	NS4A	2196	2210
MFLGGVKPTHISYIM	NS4A	2201	2215
VKPTHISYVMLIFFV	NS4A	2206	2220
ISYVMLIFFVLMVVV	NS4A	2211	2225
LIFFVLMVVVIPEPG	NS4A	2216	2230
AYLIIGILTLVSAVA	2K	2241	2255
GILTLVSVVAANELG	2K	2245	2259
GAAWTVYVGIVTMLSPML	NS4B	2293	2310
GIVTMLSPMLHHWIK	NS4B	2301	2315
HHWIKVEYGNLSLSG	NS4B	2311	2325
LSLSGIAQSASVLSFMDKG	NS4B	2321	2339
LSGIAQSASVLSFMDKGI	NS4B	2323	2340
SVLSFMDKGIPFMKM	NS4B	2331	2345
MDKGIPFMKMNISVI	NS4B	2336	2350
PFMKMNISVIMLLVS	NS4B	2341	2355
NISVIMLLVSGWNSI	NS4B	2346	2360
MLLVSGWNSITVMPLLCGI	NS4B	2351	2369
LCGIGCAMLHWSLIL	NS4B	2366	2380
CAMLHWSLILPGIKA	NS4B	2371	2385

WSLILPGIKAQQSKLAQR	NS4B	2376	2393
SLILPGIKAQQSKLAQRRV	NS4B	2377	2395
AQRRVFHGVAKNPVV	NS4B	2391	2405
ALYEKKLALYLLLAL	NS4B	2421	2435
KLALYLLLALSLASV	NS4B	2426	2440
LLLALSLASVAMCRT	NS4B	2431	2445
SLASVAMCRTPFSLA	NS4B	2436	2450
CRTPFSLAEGIVLASAAL	NS4B	2443	2460
EGIVLASAALGPLIE	NS4B	2451	2465
GNTSLLWNGPMAVSM	NS4B	2466	2480
GNYYAFVGVMYNLWK	NS4B	2486	2500
YNLWKMKTGRRGSAN	NS4B	2496	2510
AFVGVMYNLWKMKTGRR	NS4B	2490	2506
TLGEVWKRELNLLDKRQ	NS5	2513	2529
DKRQFELYKRTDIVE	NS5	2526	2540
ELYKRTDIVEVDRDTAR	NS5	2531	2547
FHERGYVKLEGRVIDLGCG	NS5	2571	2589
ERGYVKLEGRVIDLGCGR	NS5	2573	2590
GGWCYYAAAQREVSG	NS5	2591	2605
HEKPMNVQSLGWNII	NS5	2616	2630
NVQSLGWNIITFKDK	NS5	2621	2635
KDKTDIHRLEPVKCDTL	NS5	2633	2649
GVDNFCVKVLAPYMP	NS5	2681	2695
YMPDVLEKLELLQRRFGG	NS5	2693	2710
STHEMYYVSGARSNV	NS5	2721	2735
YYVSGARSNVTFTVN	NS5	2726	2740
ARSNVTFTVNQTSRL	NS5	2731	2745
TFTVNQTSRLLMRRM	NS5	2736	2750
QTSRLLMRRMRRPTGKVTLE	NS5	2741	2760
QTSRLLMRRMRRPTGKV	NS5	2741	2757
ADVILPIGTRSVETD	NS5	2761	2775
ERVERIKSEYTATWF	NS5	2781	2795
PYRTWHYCGSYVTKT	NS5	2806	2820
SMVNGVIKILTYPWD	NS5	2826	2840
VIKILTYPWDRIEEVTR	NS5	2831	2847
EEVTRMAMTDTTPFGQQ	NS5	2843	2859
PPAGTRKIMKVVNRWLFR	NS5	2873	2890
MKVVNRWLFRHLARE	NS5	2881	2895
NRWLFRHLAREKNPRLC	NS5	2885	2901
CTKEEFIAKVRSHAAIGAY	NS5	2901	2919
KEEFIAKVRSHAAIGAYL	NS5	2903	2920
QGRCRTCVYNMMGKREK	NS5	2951	2967
GKREKKLSEFGKAKGSR	NS5	2963	2979
AKGSRAIWYMWLGAR	NS5	2975	2989
GARYLEFEALGFLNEDH	NS5	2987	3003
GIGLQYLGYVIRDLAAMD	NS5	3016	3033
IGLQYLGYVIRDLAAMDGG	NS5	3017	3035
GGFYADDTAGWDTRITE	NS5	3035	3051
DLDDEQEILNYMSPHHKK	NS5	3053	3070
LNYMSPHHKKLAQAV	NS5	3061	3075
LAQAVMEMTYKNKVV	NS5	3071	3085

MEMTYKNKVVKVLRP	NS5	3076	3090
KNKVVKVLRPAPGGK	NS5	3081	3095
KAYMDVISRRDQRGSGQ	NS5	3095	3111
QVVTYALNTITNLKV	NS5	3111	3125
ALNTITNLKVQLIRM	NS5	3116	3130
TNLKVQLIRMAEAEM	NS5	3121	3135
VQLIRMAEAEMVIHHQH	NS5	3125	3141
QDCDESVLTRLEAWLTE	NS5	3143	3159
DDCVVRPIDDRFGLALS	NS5	3173	3189
DDRFGLALSHLNAMS	NS5	3181	3195
LALSHLNAMSKVRKD	NS5	3186	3200
HHFHELQLKDGRRIV	NS5	3221	3235
LQLKDGRRIVVPCRE	NS5	3226	3240
WMIKETACLSKAYAN	NS5	3256	3270
CLSKAYANMWSLMYFHKR	NS5	3263	3280
MWSLMYFHKRDMRLL	NS5	3271	3285
YFHKRDMRLLSLAVS	NS5	3276	3290
DMRLLSLAVSSAVPT	NS5	3281	3295
EVWNRVWITNNPHMQ	NS5	3321	3335
LCGSLIGMTNRATWA	NS5	3356	3370
RATWASHIHLVIHRI	NS5	3366	3380
SHIHLVIHRIRTLIGQEKYT	NS5	3371	3390
SHIHLVIHRIRTLIGQE	NS5	3371	3387
DYLTVMDRYSVDADL	NS5	3391	3405

Table S4. List of all YFV peptides identified

	Sequence	Protein	Start	End	HLA restriction	Positive/tested	Total SFC (10^6 PBMCs)
1	GFIFFFLFNILTGKK	C	46	60	DRB1*01:01	3/3	933
2	FLFNILTGKKITAHL	C	51	65	DRB1*01:01	3/3	1413
3	LRKVKRVVASLMRGL	C	81	95	DRB1*01:01	1/3	113
4	FVRNPFFAVTALTIA	M	246	260	DRB1*01:01	1/3	307
5	FFAVTALTIAYLVGS	M	251	265	DRB1*01:01	1/3	113
6	CAKFTCAKSMSLFEV	E	401	415	DRB1*01:01	1/3	207
7	NNLYKLHGGHVSCRV	E	556	570	DRB1*01:01	3/3	2287
8	VSCRVKLSALTLKGT	E	566	580	DRB1*01:01	1/3	133
9	WSPRERLVLTLGAAM	NS2A	1226	1240	DRB1*01:01	1/3	367
10	RLVLTLGAAMVEIAL	NS2A	1231	1245	DRB1*01:01	1/3	167
11	GLWKYLNAVSLCILT	NS2A	1251	1265	DRB1*01:01	1/3	280
12	LCILTINAVASRKAS	NS2A	1261	1275	DRB1*01:01	1/3	87
13	AALHPFALLLVLAGW	NS2B	1461	1475	DRB1*01:01	1/3	193
14	FALLLVLAGWLFHVR	NS2B	1466	1480	DRB1*01:01	1/3	320
15	HATLTYRMLEPTRVV	NS3	1751	1765	DRB1*01:01	1/3	907
16	YRMLEPTRVVNWEVI	NS3	1756	1770	DRB1*01:01	1/3	847
17	AHHVCWLEASMLLDN	NS3	1971	1985	DRB1*01:01	3/3	740
18	MDKGIPFMKMNISVI	NS4B	2336	2350	DRB1*01:01	1/3	97
19	ALYEKKLALYLLLAL	NS4B	2421	2435	DRB1*01:01	1/3	173
20	EGIVLASAALGPLIE	NS4B	2451	2465	DRB1*01:01	1/3	667
21	GNTSLLWNGPMAVSM	NS4B	2466	2480	DRB1*01:01	1/3	2027
22	FHERGYVKLEGRVID	NS5	2571	2585	DRB1*01:01	1/3	347
23	FIAKVRSHAAIGAYL	NS5	2906	2920	DRB1*01:01	1/3	387
24	IWYMWLGARYLEFEA	NS5	2981	2995	DRB1*01:01	1/3	737
25	LCGSLIGMTNRATWA	NS5	3356	3370	DRB1*01:01	1/3	87
26	GTVVMQVKVSKGAPC	E	601	615	DRB1*03:01	1/2	93
27	RIPVIVADDLTAAIN	E	616	630	DRB1*03:01	1/2	93
28	VVVQDPKNVYQRGTH	NS1	866	880	DRB1*03:01	2/2	267
29	FTTRVYMDAVFEYTI	NS1	941	955	DRB1*03:01	1/2	133
30	GHDWILADKRPTAWF	NS3	1836	1850	DRB1*03:01	1/2	293
31	MRLRDDQRKVFRELV	NS3	2011	2025	DRB1*03:01	1/2	140
32	EEHEILNDSGETVKC	NS3	2056	2070	DRB1*03:01	1/2	393
33	QQRSIQDNQVAYLII	NS4B	2231	2245	DRB1*03:01	1/2	93
34	YLGYVIRDLAAMDGG	NS5	3021	3035	DRB1*03:01	1/2	193
35	GFIFFFLFNILTGKK	C	46	60	DRB1*04:01	2/5	893
36	RIPVIVADDLTAAIN	E	616	630	DRB1*04:01	1/5	280
37	LIWVGINTRNMTMSM	E	746	760	DRB1*04:01	2/5	1207
38	LLMMLVSVAGRVDGL	NS2B	1391	1405	DRB1*04:01	1/5	320
39	ILVGDNSFVSAISQT	NS3	1641	1655	DRB1*04:01	2/5	487
40	NSFVSAISQTEVKEE	NS3	1646	1660	DRB1*04:01	1/5	373
41	GKEELQEIPTMLKKG	NS3	1661	1675	DRB1*04:01	1/5	520
42	ARRRLRTLVLAPTRV	NS3	1701	1715	DRB1*04:01	1/5	620
43	RTLVLAPTRVVLSEM	NS3	1706	1720	DRB1*04:01	1/5	473
44	KEAFHGLDVKFHTQA	NS3	1721	1735	DRB1*04:01	1/5	287

45	LPSIRAANVMAASLR	NS3	1851	1865	DRB1*04:01	3/5	700
46	IKGPLRISASSAAQR	NS3	1931	1945	DRB1*04:01	1/5	527
47	DGDSYYYSEPTSENN	NS3	1956	1970	DRB1*04:01	3/5	1610
48	DERVSSDQSALSEFI	NS3	2086	2100	DRB1*04:01	1/5	460
49	EEGSRAYRNALSMMP	NS4A	2141	2155	DRB1*04:01	1/5	433
50	ISYIMLIFFVLMVVV	NS4A	2211	2225	DRB1*04:01	1/5	147
51	WTVYVGIVTMLSPML	NS4B	2296	2310	DRB1*04:01	1/5	360
52	LSLSGIAQSASVLSF	NS4B	2321	2335	DRB1*04:01	1/5	353
53	PGIKAQQSKLAQRRV	NS4B	2381	2395	DRB1*04:01	1/5	100
54	EGIVLASAALGPLIE	NS4B	2451	2465	DRB1*04:01	1/5	253
55	MTSWFYDNDNPYRTW	NS5	2796	2810	DRB1*04:01	1/5	147
56	DEQEILNYMSPHHKK	NS5	3056	3070	DRB1*04:01	3/5	743
57	ALNTITNLKVQLIRM	NS5	3116	3130	DRB1*04:01	1/5	107
58	VRRGVRSLSNKIKQK	C	16	30	DRB1*04:03	1/3	3013
59	LRKVKRVVASLMRGL	C	81	95	DRB1*04:03	1/3	707
60	RVVASLMRGLSSRKR	C	86	100	DRB1*04:03	1/3	200
61	TDKMFFVKNPTDTGH	E	586	600	DRB1*04:03	1/3	113
62	VSMMIAMEVVLRKRQ	NS2A	1141	1155	DRB1*04:03	1/3	320
63	NTILPLMALLTPVTM	NS2A	1276	1290	DRB1*04:03	1/3	160
64	PTAWFLPSIRAANVM	NS3	1846	1860	DRB1*04:03	1/3	100
65	LPSIRAANVMAASLR	NS3	1851	1865	DRB1*04:03	1/3	193
66	SLASVAMCRTPFSLA	NS4B	2436	2450	DRB1*04:03	1/3	967
67	FIAKVRSHAAIGAYL	NS5	2906	2920	DRB1*04:03	2/3	507
68	VRKVCYNAVLTHVKI	E	341	355	DRB1*04:07	3/3	1540
69	CAKFTCAKSMSLFEV	E	401	415	DRB1*04:07	1/3	127
70	TDKMFFVKNPTDTGH	E	586	600	DRB1*04:07	1/3	273
71	GLWKYLNAVSLCILT	NS2A	1251	1265	DRB1*04:07	1/3	153
72	RFLPQILAECARRRL	NS3	1691	1705	DRB1*04:07	2/3	2393
73	PTAWFLPSIRAANVM	NS3	1846	1860	DRB1*04:07	1/3	567
74	LPSIRAANVMAASLR	NS3	1851	1865	DRB1*04:07	1/3	280
75	EEGSRAYRNALSMMP	NS4A	2141	2155	DRB1*04:07	1/3	233
76	AYRNALSMMPEAMTI	NS4A	2146	2160	DRB1*04:07	1/3	260
77	SLASVAMCRTPFSLA	NS4B	2436	2450	DRB1*04:07	2/3	1053
78	FIAKVRSHAAIGAYL	NS5	2906	2920	DRB1*04:07	1/3	233
79	KAYANMWSLMYFHKR	NS5	3266	3280	DRB1*04:07	1/3	513
80	DMRLLSLAVSSAVPT	NS5	3281	3295	DRB1*04:07	1/3	103
81	GFIFFFLFNILTGKK	C	46	60	DRB1*07:01	1/3	87
82	DQTKIQYVIRAQLHV	E	416	430	DRB1*07:01	1/3	227
83	LPSIRAANVMAASLR	NS3	1851	1865	DRB1*07:01	1/3	110
84	IKGPLRISASSAAQR	NS3	1931	1945	DRB1*07:01	1/3	430
85	DMRLLSLAVSSAVPT	NS5	3281	3295	DRB1*07:01	1/3	2100
86	VRRGVRSLSNKIKQK	C	16	30	DRB1*08:02	1/1	620
87	QGLAVLRKVKRVVAS	C	76	90	DRB1*08:02	1/1	700
88	GVTLVRKNRWLLLNV	M	121	135	DRB1*08:02	1/1	87
89	GHDWILADKRPTAWF	NS3	1836	1850	DRB1*08:02	1/1	320
90	QTSRLLMRRMRRPTG	NS5	2741	2755	DRB1*08:02	1/1	800
91	CTKEEFIAKVRSHAA	NS5	2901	2915	DRB1*08:02	1/1	467

92	VRKVCYNAVLTHVKI	E	341	355	DRB1*10:01	1/1	520
93	CAKFTCAKSMSLFEV	E	401	415	DRB1*10:01	1/1	207
94	LIWVGINTRNMTMSM	E	746	760	DRB1*10:01	1/1	107
95	LCILTINAVASRKAS	NS2A	1261	1275	DRB1*10:01	1/1	140
96	NTILPLMALLTPVTM	NS2A	1276	1290	DRB1*10:01	1/1	160
97	AGKTRRFLPQILAEC	NS3	1686	1700	DRB1*10:01	1/1	673
98	RFLPQILAECARRRL	NS3	1691	1705	DRB1*10:01	1/1	1087
99	HATLTYRMLEPTRVV	NS3	1751	1765	DRB1*10:01	1/1	307
100	PTAWFLPSIRAANVM	NS3	1846	1860	DRB1*10:01	1/1	187
101	HHWIKVEYGNLSLSG	NS4B	2311	2325	DRB1*10:01	1/1	507
102	SLASVAMCRTPFSLA	NS4B	2436	2450	DRB1*10:01	1/1	287
103	QVVTYALNTITNLKV	NS5	3111	3125	DRB1*10:01	1/1	87
104	WMIKETACLSKAYAN	NS5	3256	3270	DRB1*10:01	1/1	293
105	GFIFFFLFNILTGKK	C	46	60	DRB1*11:01	1/4	200
106	FLFNILTGKKITAHL	C	51	65	DRB1*11:01	1/4	160
107	ITAHLKRLWKMLDPR	C	61	75	DRB1*11:01	1/4	87
108	QGLAVLRKVKRVVAS	C	76	90	DRB1*11:01	1/4	93
109	LRKVKRVVASLMRGL	C	81	95	DRB1*11:01	1/4	180
110	PPHAATIRVLALGNQ	E	521	535	DRB1*11:01	1/4	213
111	GTVVMQVKVSKGAPC	E	601	615	DRB1*11:01	1/4	233
112	FGDSYIIVGRGDSRL	E	656	670	DRB1*11:01	1/4	233
113	GVIMMFLSLGVGADQ	E	766	780	DRB1*11:01	1/4	573
114	IAVGGLLMMLVSVAG	NS2B	1386	1400	DRB1*11:01	1/4	87
115	LLMMLVSVAGRVDGL	NS2B	1391	1405	DRB1*11:01	1/4	147
116	GAFLVRNGKKLIPSW	NS3	1541	1555	DRB1*11:01	1/4	300
117	GHDWILADKRPTAWF	NS3	1836	1850	DRB1*11:01	1/4	200
118	EEGSRAYRNALSMMP	NS4A	2141	2155	DRB1*11:01	1/4	150
119	FVGVMYNLWKMKTGR	NS4B	2491	2505	DRB1*11:01	1/4	293
120	QTSRLLMRRMRRPTG	NS5	2741	2755	DRB1*11:01	2/4	1443
121	LMRRMRRPTGKVTLE	NS5	2746	2760	DRB1*11:01	2/4	1633
122	DMRLLSLAVSSAVPT	NS5	3281	3295	DRB1*11:01	1/4	227
123	VIHRIRTLIGQEKYT	NS5	3376	3390	DRB1*11:01	1/4	113
124	ITAHLKRLWKMLDPR	C	61	75	DRB1*11:04	3/4	2313
125	QGLAVLRKVKRVVAS	C	76	90	DRB1*11:04	1/4	600
126	LRKVKRVVASLMRGL	C	81	95	DRB1*11:04	2/4	3587
127	VRKVCYNAVLTHVKI	E	341	355	DRB1*11:04	2/4	333
128	THVKINDKCPSTGEA	E	351	365	DRB1*11:04	3/4	553
129	GSIVACAKFTCAKSM	E	396	410	DRB1*11:04	3/4	1760
130	PPHAATIRVLALGNQ	E	521	535	DRB1*11:04	1/4	93
131	FGDSYIIVGRGDSRL	E	656	670	DRB1*11:04	1/4	247
132	LPSIRAANVMAASLR	NS3	1851	1865	DRB1*11:04	1/3	153
133	KAGKSVVVLNRKTFE	NS3	1866	1880	DRB1*11:04	2/3	593
134	VVVLNRKTFEREYPT	NS3	1871	1885	DRB1*11:04	3/3	733
135	PFMKMNISVIMLLVS	NS4B	2341	2355	DRB1*11:04	1/3	187
136	SLASVAMCRTPFSLA	NS4B	2436	2450	DRB1*11:04	1/3	223
137	FVGVMYNLWKMKTGR	NS4B	2491	2505	DRB1*11:04	1/3	1607
138	RTVRVLDTVEKWLAC	NS5	2666	2680	DRB1*11:04	1/3	173

139	QTSRLLMRRMRRPTG	NS5	2741	2755	DRB1*11:04	2/3	1690
140	LMRRMRRPTGKVTLE	NS5	2746	2760	DRB1*11:04	3/3	2753
141	SHIHLVIHRIRTLIG	NS5	3371	3385	DRB1*11:04	1/3	113
142	VIHRIRTLIGQEKYT	NS5	3376	3390	DRB1*11:04	1/3	213
143	IGKLFTQTMKGVERL	E	681	695	DRB1*12:01	1/2	140
144	PVWLSWQVAKAGLKT	NS3	2031	2045	DRB1*12:01	1/2	160
145	GVDNFCVKVLAPYMP	NS5	2681	2695	DRB1*12:01	1/2	853
146	LGVNMVRRGVRSLSN	C	11	25	DRB1*13:01	2/5	880
147	KRLWKMLDPRQGLAV	C	66	80	DRB1*13:01	1/5	180
148	QGLAVLRKVKRVVAS	C	76	90	DRB1*13:01	4/5	1500
149	RVVASLMRGLSSRKR	C	86	100	DRB1*13:01	1/5	1887
150	GVTLVRKNRWLLLNV	M	121	135	DRB1*13:01	2/5	2033
151	ERQLQKIERWFVRNP	M	236	250	DRB1*13:01	1/5	233
152	GSIVACAKFTCAKSM	E	396	410	DRB1*13:01	2/5	593
153	GTVVMQVKVSKGAPC	E	601	615	DRB1*13:01	2/5	500
154	LIWVGINTRNMTMSM	E	746	760	DRB1*13:01	1/5	477
155	EVDISVVVQDPKNVY	NS1	861	875	DRB1*13:01	3/5	1957
156	VVVQDPKNVYQRGTH	NS1	866	880	DRB1*13:01	3/5	1857
157	SHLVRSWVTAGEIHA	NS1	1121	1135	DRB1*13:01	1/5	107
158	VSMMIAMEVVLRKRQ	NS2A	1141	1155	DRB1*13:01	2/5	633
159	NNGGDAMYMALIAAF	NS2A	1196	1210	DRB1*13:01	2/5	2523
160	LLIGFGLRTLWSPRE	NS2A	1216	1230	DRB1*13:01	1/5	1873
161	TPVTMAEVRLAAMFF	NS2A	1286	1300	DRB1*13:01	1/5	87
162	GAFLVRNGKKLIPSW	NS3	1541	1555	DRB1*13:01	1/5	1000
163	PTRVVNWEVIIMDEA	NS3	1761	1775	DRB1*13:01	1/5	700
164	KAGKSVVVLNRKTFE	NS3	1866	1880	DRB1*13:01	4/5	2180
165	VVVLNRKTFEREYPT	NS3	1871	1885	DRB1*13:01	4/5	4140
166	EVWKRELNLLDKRQF	NS5	2516	2530	DRB1*13:01	1/5	353
167	QTSRLLMRRMRRPTG	NS5	2741	2755	DRB1*13:01	1/5	173
168	LMRRMRRPTGKVTLE	NS5	2746	2760	DRB1*13:01	1/5	320
169	RWLFRHLAREKNPRL	NS5	2886	2900	DRB1*13:01	3/5	1140
170	TNLKVQLIRMAEAEM	NS5	3121	3135	DRB1*13:01	1/5	113
171	MWSLMYFHKRDMRLL	NS5	3271	3285	DRB1*13:01	2/5	613
172	DMRLLSLAVSSAVPT	NS5	3281	3295	DRB1*13:01	1/5	363
173	SHIHLVIHRIRTLIG	NS5	3371	3385	DRB1*13:01	1/5	313
174	VRRGVRSLSNKIKQK	C	16	30	DRB1*15:01	4/8	3127
175	GVTLVRKNRWLLLNV	M	121	135	DRB1*15:01	1/8	573
176	GVIMMFLSLGVGADQ	E	766	780	DRB1*15:01	1/8	740
177	SIRPGLLIGFGLRTL	NS2A	1211	1225	DRB1*15:01	1/8	473
178	LLIGFGLRTLWSPRE	NS2A	1216	1230	DRB1*15:01	1/8	360
179	GGVMGGLWKYLNAVS	NS2A	1246	1260	DRB1*15:01	1/8	320
180	TQPFLGLCAFLATRI	NS2A	1336	1350	DRB1*15:01	1/8	770
181	DGIYGIFQSTFLGAS	NS3	1506	1520	DRB1*15:01	1/8	1053
182	GEVIGLYGNGILVGD	NS3	1631	1645	DRB1*15:01	3/8	3393
183	TSGMVIFFMSPKGIS	NS4A	2171	2185	DRB1*15:01	1/8	160
184	ISYIMLIFFVLMVVV	NS4A	2211	2225	DRB1*15:01	1/8	613
185	LIFFVLMVVVIPEPG	NS4A	2216	2230	DRB1*15:01	1/8	87

186	KPGAAWTVYVGIVTM	NS4B	2291	2305	DRB1*15:01	1/8	127
187	SMVNGVIKILTYPWD	NS5	2826	2840	DRB1*15:01	1/8	173
188	VIKILTYPWDRIEEV	NS5	2831	2845	DRB1*15:01	1/8	243
189	GTRKIMKVVNRWLFR	NS5	2876	2890	DRB1*15:01	3/8	1720
190	DEQEILNYMSPHHKK	NS5	3056	3070	DRB1*15:01	1/8	200
191	GFIFFFLFNILTGKK	C	46	60	DRB1*15:02	1/2	113
192	KIERWFVRNPFFAVT	M	241	255	DRB1*15:02	1/2	1640
193	FVRNPFFAVTALTIA	M	246	260	DRB1*15:02	1/2	147
194	GEVIGLYGNGILVGD	NS3	1631	1645	DRB1*15:02	1/2	363
195	GCGYLMFLGGVKPTH	NS4A	2196	2210	DRB1*15:02	1/2	270
196	LIFFVLMVVVIPEPG	NS4A	2216	2230	DRB1*15:02	1/2	203
197	GWNSITVMPLLCGIG	NS4B	2356	2370	DRB1*15:02	1/2	117
198	ALYEKKLALYLLLAL	NS4B	2421	2435	DRB1*15:02	1/2	163
199	KLALYLLLALSLASV	NS4B	2426	2440	DRB1*15:02	1/2	83
200	DKRQFELYKRTDIVE	NS5	2526	2540	DRB1*15:02	1/2	170
201	QTSRLLMRRMRRPTG	NS5	2741	2755	DRB1*15:02	1/2	1207
202	LMRRMRRPTGKVTLE	NS5	2746	2760	DRB1*15:02	2/2	1623
203	QVVTYALNTITNLKV	NS5	3111	3125	DRB1*15:02	1/2	110
204	GIVTMLSPMLHHWIK	NS4B	2301	2315	DRB1*15:06	1/1	250
205	DEQEILNYMSPHHKK	NS5	3056	3070	DRB1*15:06	1/1	570
206	VRRGVRSLSNKIKQK	C	16	30	DRB1*16:02	1/1	573
207	KIERWFVRNPFFAVT	M	241	255	DRB1*16:02	1/1	320
208	CAKFTCAKSMSLFEV	E	401	415	DRB1*16:02	1/1	180
209	QYVIRAQLHVGAKQE	E	421	435	DRB1*16:02	1/1	1270
210	PKNVYQRGTHPFSRI	NS1	871	885	DRB1*16:02	1/1	1600
211	VSMMIAMEVVLRKRQ	NS2A	1141	1155	DRB1*16:02	1/1	290
212	GAFLVRNGKKLIPSW	NS3	1541	1555	DRB1*16:02	1/1	113
213	ILAECARRRLRTLVL	NS3	1696	1710	DRB1*16:02	1/1	4983
214	GNHYAFVGVMYNLWK	NS4B	2486	2500	DRB1*16:02	1/1	140
215	MKVVNRWLFRHLARE	NS5	2881	2895	DRB1*16:02	1/1	480
